# Role of Radiomics to Predict Disease Recurrence in Sinonasal Squamous Cell Carcinoma: A Systematic Review and Meta‐Analysis

**DOI:** 10.1111/coa.70031

**Published:** 2025-09-05

**Authors:** Caitlin Waters, Tamzin Hall, Hugo C. Temperley, Holly Jones, Niall J. O'Sullivan, Alison McHugh, Fariba Tohidinezhad, Thavakumar Subramaniam

**Affiliations:** ^1^ Department of Otolaryngology—Head and Neck Surgery Galway University Hospital Galway Ireland; ^2^ Sir Charles Gairdner Hospital Perth Western Australia Australia; ^3^ Department of Radiology St. James's Hospital Dublin Ireland; ^4^ Trinity St James Cancer Institute Trinity College Dublin Dublin Ireland; ^5^ Department of Radiology and Nuclear Medicine Erasmus University Medical Center Rotterdam Rotterdam the Netherlands

**Keywords:** oncology, radiogenomics, radiomics, recurrence, sinonasal cancer

## Abstract

**Introduction:**

Radiomics offers the potential to predict oncological outcomes from pre‐operative imaging, aiding in the identification of ‘high risk’ patients with sinonasal cancer who are at an increased risk of recurrence. This study aims to comprehensively review the current literature on the role of radiomics as a predictor of disease recurrence in sinonasal squamous cell carcinoma.

**Methods:**

A systematic search was conducted in Medline, EMBASE and Web of Science databases. Retrospective and prospective studies examining the use of radiomics to predict post‐operative recurrence in sinonasal cancer that met the inclusion criteria were included. Study quality was assessed using the QUADAS‐2 and Radiomics Quality Score (RQS) tools.

**Results:**

Five studies met the inclusion criteria, encompassing 638 participants. All studies were single‐centre and utilised MRI‐based radiomics in the construction of their models. Radiomic models demonstrated excellent predictive performance. The median AUC, sensitivity and specificity were 0.947, 0.86 and 0.923 in the training set, and 0.914, 0.833 and 0.878 in the validation set. A pooled meta‐analysis estimated the combined AUC across training sets as 0.931 (95% CI, 0.898–0.963) and 0.922 (95% CI, 0.880–0.964) for validation sets.

**Conclusion:**

Our systematic review provides evidence supporting the role of radiomics in predicting post‐operative disease recurrence in sinonasal cancer. Radiomics shows promise in enhancing personalised treatment strategies by improving prognostic accuracy. However, further research is needed to standardise methodologies and validate these findings in larger, multicentre cohorts.

**Summary**

- Radiomics enables pre‐operative imaging to predict recurrence risk in sinonasal squamous cell carcinoma.
- This is the first systematic review to comprehensively evaluate radiomics as a predictor of recurrence in sinonasal squamous cell carcinoma.
- Radiomic models showed excellent predictive performance with pooled AUCs of 0.931 (training) and 0.922 (validation).
- Radiomics holds promise for improving personalised treatment but requires further multicentre validation.
- Integrating radiomics with molecular or genetic biomarkers could enhance prognostic accuracy.

## 1 Introduction

Sinonasal squamous cell carcinoma (SNSCC) is a relatively rare malignancy, accounting for approximately 3%–5% of all head and neck cancers, with an incidence of 0.556 cases per 100 000 population per year [1, 2, 3]. Despite curative‐intent surgery, a substantial number of patients with sinonasal cancer experience disease recurrence and poor prognosis, highlighting the need for novel prognostic tools to enhance risk stratification and guide treatment decisions [2, 4]. Radiomics encompasses the high‐throughput extraction and analysis of a multitude of quantitative features from medical images, such as computed tomography (CT) scans, magnetic resonance imaging (MRI) and positron emission tomography (PET) [5]. The application of radiomics in predicting disease recurrence is particularly pertinent, as it may enable a more precise and individualised assessment of post‐operative risk, allowing for tailored therapeutic strategies [5, 6, 7].

Radiomics offers the potential to predict oncological outcomes from pre‐operative imaging in order to identify ‘high risk’ patients at increased risk of recurrence [8]. The performance of radiomics signatures may be improved further when combined with clinicopathological data in the form of a nomogram, which could be used as a prognostic tool to enhance shared decision‐making and patient counselling as a whole [9, 10]. Such personalised risk assessment tools could facilitate the identification of high‐risk patients who might benefit from intensified adjuvant therapies or closer post‐operative monitoring [6, 11, 12, 13]. Conversely, low‐risk patients may be spared unnecessary interventions, thereby reducing treatment‐related morbidity and healthcare costs [14]. To the best of the authors' knowledge, this is the first systematic review to comprehensively assess the existing literature regarding the role of radiomics as a predictor of disease recurrence in SNSCC.

## 2 Methods

### 2.1 Study Design and Reporting Guidelines

This study is a systematic review of non‐randomised trials and follows the Preferred Reporting Items for Systematic Reviews and Meta‐Analyses (PRISMA) reporting guidelines [15].

The methodology employed in this study was similar to that used in our group's previous radiomics studies, with necessary modifications to address the specific aims and dataset related to SNSCC [6, 13, 16, 17].

### 2.2 Search Strategy

The following databases were searched as part of the systematic review in May 2024: Medline, EMBASE and Web of Science. The systematic search process with detailed search terms is outlined in Supporting Information S1. The last date of search was 5 May 2024. The grey literature was also searched to further identify other suitable publications.

### 2.3 Inclusion Criteria

Studies in English were assessed for eligibility based on the following inclusion criteria: studies investigating the use of radiomics to predict post‐operative recurrence in patients with sinonasal cancer were included in our analysis. Case reports, case series and conference abstracts were excluded.

- Study design:
  - Cohort studies.
  - Original research (> 20 patients).
- Participants:
  - Patients with sinonasal cancer.
- Intervention:
  - Radiomic signature development.
- Outcomes:
  - Oncological outcomes: recurrence, disease‐free survival, overall survival, pathological complete response.

### 2.4 Study Selection, Data Extraction and Critical Appraisal

A database was created using the reference managing software EndNote X9. Two researchers (C.W. and N.J.O.) reviewed outputs from the searches independently of each other.

Initially, duplicates were removed. Study titles were then screened and assessed for potential relevance. The abstracts of selected potential studies were then read and assessed for eligibility for inclusion, based on the inclusion/exclusion criteria detailed above. Rejected studies were grouped together in the database by their reason for exclusion. The full texts of the abstracts deemed eligible for inclusion were then further analysed using the same criteria [16].

In order to extract and store data efficiently, the Cochrane Collaboration screening and data extraction tool, Covidence, was used [18]. Data was collected by two reviewers (C.W. and N.J.O.) independently, using the following headings: study details, study design, population, intervention, comparison groups and outcomes. Conflicts on study selection and data extraction between the two reviewers (C.W. and N.J.O.) were resolved following an open discussion and a final decision [17].

A critical appraisal of the methodological quality and risk of bias of the included studies was performed. The critical appraisal was completed by two reviewers independently. Quality assessment of the included studies was performed according to the Quality Assessment of Diagnostic Accuracy Studies 2 (QUADAS‐2) and Radiomics Quality Score (RQS) [13, 19, 20].

### 2.5 Statistical Analysis

A pooled meta‐analysis of means was conducted using a random‐effects model to account for heterogeneity amongst studies. The weighted mean area under the curve (AUC) values and their corresponding 95% confidence intervals (CI) were calculated, with weights assigned based on the inverse variance of each study's AUC estimate.

### 2.6 Systematic Review Registration

Our systematic review was registered on PROSPERO in March 2025 (ID: CRD420250577671) [21].

## 3 Results

### 3.1 Search Results

**FIGURE 1:**
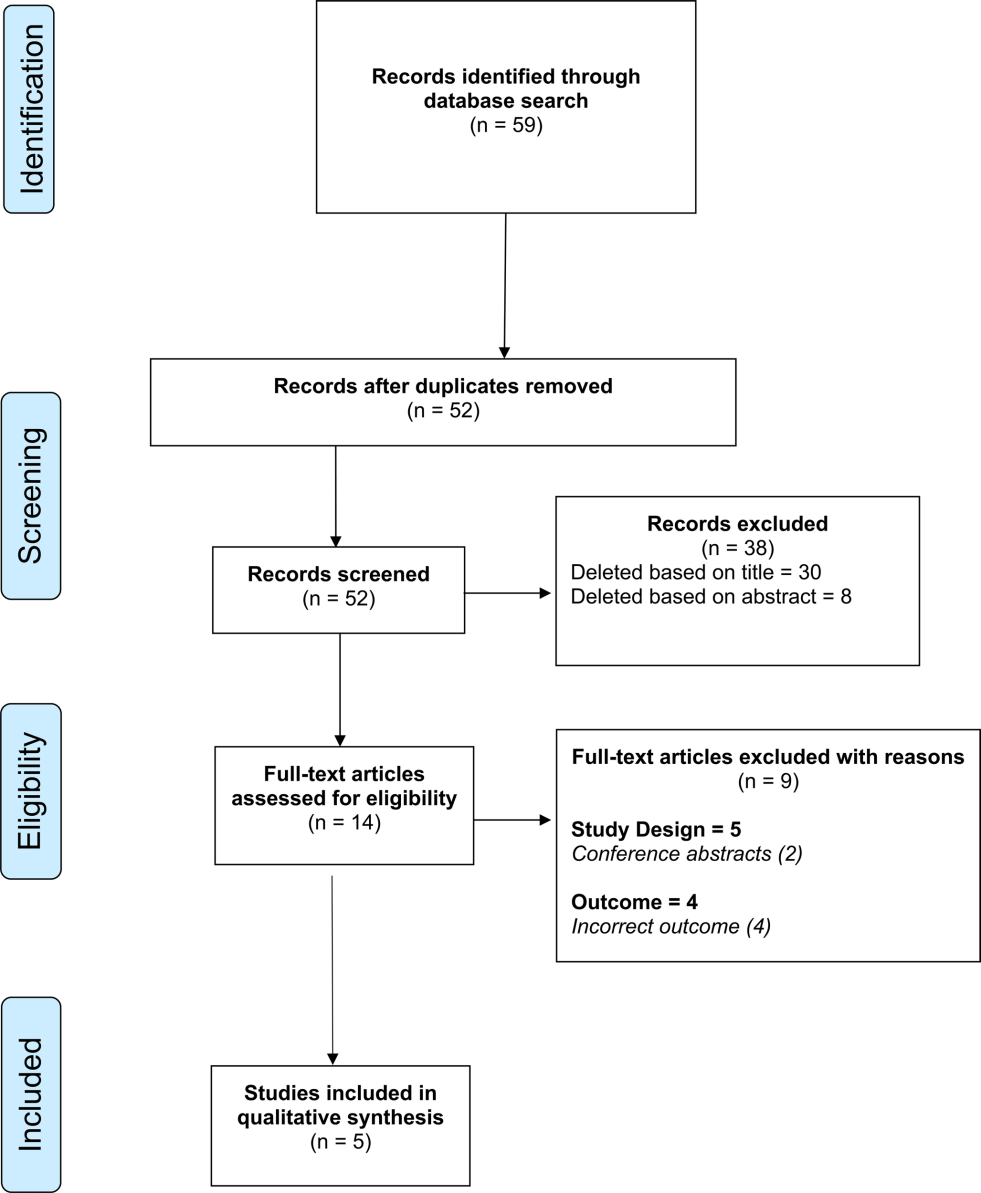
Study selection. A PRISMA flowchart of the selection of relevant publications included in this review.

The literature search described above yielded a total of 59 results (Figure 1). Following the removal of 7 duplicates, 52 studies were screened. After the initial screen, 14 abstracts were reviewed and assessed for eligibility, of which 5 were selected for full text review. From these five full texts, a total of five studies met the inclusion criteria and were included in our analysis [22, 23, 24, 25, 26].

### 3.2 Methodological Characteristics and Quality of Studies

Overall, four of the five studies were retrospective in nature, with one study being prospective and all studies being single centre [22, 23, 24, 25, 26]. All the studies included in the review were published in Asian countries, with three originating from China. Table 1 summarises the methodological characteristics of the included studies. Data quality, assessed using the RQS and QUADAS‐2 tools, was generally satisfactory [19, 20]. All studies were deemed low risk of bias as assessed by the QUADAS‐2 tool and received an RQS score > 30% [19, 20]. A detailed explanation of the tools and breakdown of the results can be found in Supporting Information S1–S4.

**TABLE 1:** Methodological characteristics of the included studies.

Study	Country	Journal	Study design	Single centre	Primary outcome
Fujima et al. (2019) [22]	Japan	*Cancers*	Retrospective	Yes	Prediction of treatment outcome (local control and local failure)
Park et al. (2024) [26]	South Korea	*European Radiology*	Retrospective	Yes	Prediction of early local failure
N. Lin et al. (2022) [25]	China	*Frontiers in Oncology*	Prospective	Yes	Prediction of risk of early relapse
Geng et al. (2023) [23]	China	*European Archives of Oto‐Rhino‐Laryngology*	Retrospective	Yes	Prediction of histological grade
M. Lin et al. (2023) [24]	China	*Academic Radiology*	Retrospective	Yes	Prediction of the risk of 2‐year recurrence

### 3.3 Participant Characteristics

The total number of participants from the five included studies was 638 [22, 23, 24, 25, 26]. All studies included both training and validation sets within their studies, with 71.0% (*n* = 453) of patients in the training set and 29.0% (*n* = 185) of patients in the validation set [22, 23, 24, 25, 26]. Of these participants, 73.9% (*n* = 585) were male and 26.1% (*n* = 207) were female [22, 23, 24, 25, 26]. Basic participant characteristics and management details are outlined in Table 2.

**TABLE 2:** Clinical patient characteristics.

Study	No. patients		Male:female	Age (mean)		Origin of cancer	Stage of cancer	Treatment
	T	V		T	V			
Fujima et al. (2019) [22]	32	4	28:8	58.7		Nasal cavity: *n* = 6 Paranasal sinus: *n* = 30	T2: *n* = 1 T3: *n* = 13 T4a: *n* = 17 T4b: 5 N0: *n* = 30 N1: *n* = 2 N2: *n* = 4	N/A
Park et al. (2024) [26]	47	21	52:16	56.0	61.0	Maxillary sinus: *n* = 54 Ethmoid sinus + nasal cavity: *n* = 12 Sphenoid sinus: *n* = 2	T2: *n* = 7 T3: *n* = 30 T4: *n* = 31	Surgery only: *n* = 2 Surgery then adjuvant RT: *n* = 44 Neoadjuvant RT then surgery: *n* = 3 Definitive RT: *n* = 19
N. Lin et al. (2022) [25]	106	46	115:37	55.41 ± 14.59		De novo SNSCC: *n* = 106 Inverted papilloma‐derived SNSCC: *n* = 43	T1/2/3: *n* = 40 T4a/4b: *n* = 118 N0: *n* = 125 N1/2: *n* = 27 M0: *n* = 145 M1: *n* = 7	Surgery: *n* = 152 RT: *n* = 145 (95.4%) Adjuvant chemotherapy: *n* = 31 (20.4%)
Geng et al. (2023) [23]	103	44	106:41	56.4 ± 13.7		Nasal cavity: *n* = 57 Paranasal sinus: *n* = 90	TNM I‐II: *n* = 16 TNM III‐IV: *n* = 131	N/A
M. Lin et al. (2023) [24]	165	70	175:60	55.78 ± 14.67		De novo SNSCC: *n* = 191 Inverted papilloma‐derived SNSCC: *n* = 44	T1/2/3: *n* = 65 T4a/4b: *n* = 170 N0: *n* = 182 N1/2/3: *n* = 53 M0: *n* = 226 M1: *n* = 9	Surgery: *n* = 235 RT: *n* = 221 (94.0%) Pre‐operative or adjuvant chemotherapy: *n* = 58 (24.7%)

Abbreviations: ±, standard deviation; M, metastasis; N, lymph node involvement; RT, radiotherapy; SNSCC, sinonasal squamous cell carcinoma; T, tumour size.

### 3.4 Acquisition Parameters

MRI was the imaging modality utilised in all five studies, and each study used a combination of imaging sequences [22, 23, 24, 25, 26]. Acquisition parameters are illustrated in Table 3 including imaging modality, segmentation methods and imaging parameters.

**TABLE 3:** Summary of imaging parameters and radiomics.

Authors	Imaging modality	Segmentation	Selected imaging parameters	Prediction algorithm
Fujima et al. (2019) [22]	Pre‐treatment MRI (T2w, DWI)	Manual segmentation	Tumour blood flow, tumour sphericity, texture parameter of contrast, tumour volume, intermediate diffusion coefficient, perfusion fraction, apparent diffusion coefficient	Non‐linear support vector machine
Park et al. (2024) [26]	Pre‐treatment MRI (post contrast T1w, T2w)	Semiautomatic segmentation	First‐order GLCM, GLRLM, GLSZM, NGTDM	LDA, LASSO
N. Lin et al. (2022) [25]	MRI (DWI, T2w)	Manual segmentation	GLCM, GLRLM < GLSZM, NGTDM	LASSO, linear combination Multivariate logistic regression analysis?
Geng et al. (2023) [23]	Pre‐treatment MRI (T1w, T2w, DWI and contrast enhanced T1w)	Manual segmentation	Apparent diffusion coefficient	Kruskal–Wallis, logistic regression
M. Lin et al. (2023) [24]	MRI (T1c, T2w, DWI)	Automatic segmentation	GLCM, GLRLM, GLSZM, GLDM, NGTDM	LASSO and LR

Abbreviations: DWI, diffusion‐weighted imaging; GLCM, grey‐level co‐occurrence matrix, GLDM, grey‐level dependence matrix; GLRLM, grey‐level run length matrix; GLSZM, grey‐level size zone matrix; LASSO, least absolute shrinkage and selection operator; LDA, linear discriminant analysis; LR, logistic regression; MRI, magnetic resonance imaging; NGTDM, neighbourhood grey‐tone difference matrix; T1w, T1‐weighted imaging; T2w, T2‐weighted imaging.

### 3.5 Signature Development

Exact feature extraction methods varied across studies; however, a relatively similar pathway was followed across the board. Regions of interest were first segmented manually by experienced radiologists in all included studies. Radiomic features were then extracted from these segments using various radiomics software applications. All five included studies incorporated radiomics features in the development of their prognostic model. The specific software utilised in each study is demonstrated in Table 4.

**TABLE 4:** Software and performance.

Study	Segmentation software	Radiomics software	Performance of signature (training)			Performance of signature (validation)			
			AUC	Sens	Spec	AUC	Sens	Spec	Conclusions
Fujima et al. (2019) [22]	N/A	Pyradiomics 2.0	/	0.98	0.91	/	1.0	0.82	Integration of quantitative tumour morphological, intratumoral textural, perfusion and diffusion data is accurate at predicting treatment outcomes
Park et al. (2024) [26]	Medical Image Processing, Analysis, and Visualization software version 7.0	Pyradiomics 2.0	0.965 (0.901–1)	/	/	Radiomics: 0.838 (0.615–1.000) Clinical + radiomics: 0.850 (0.623–1.000)	/	/	Pre‐treatment MR imaging derived tumour data can highly predict the treatment outcome
N. Lin (2022) [25]	3D Slicer—Segment Editor module	Pyradiomics 2.0	Radscore: 0.84 (0.76–0.91) Clinical: 0.82 (0.75–0.90) Nomogram: 0.92 (0.87–0.97)	/	/	Radscore: 0.84 (0.73–0.96) Clinical: 0.79 (0.66–0.92) Nomogram: 0.92 (0.82–1.00)	/	/	MRI radiomics can predict early local failure with high accuracy
Geng et al. (2023) [23]	3D Slicer—Segment Editor module	Pyradiomics 2.0	Mean: 0.947 Clinical: 0.836 Histogram + clinical: 0.940 Mean + clinical: 0.947	/	/	Mean: 0.957 Clinical: 0.692 Histogram + clinical: 0.947 Mean + clinical: 0.957	Mean: 0.842 Clinical: 0.710 Histogram + Clinical: 0.868 Mean + Clinical: 0.842	Mean: 0.935 Clinical: 0.709 Histogram + clinical: 0.935 Mean + clinical: 0.935	MRI radiomics nomogram incorporating both radiomic features and clinical factors may predict early relapse in SNSCC
M. Lin (2023) [24]	ITK‐SNAP	uAI Research Portal	0.896 (0.740–0.975)	0.8 (0.563–0.943)	0.923 (0.640–0.998)	0.914 (0.822–0.968)	0.833 (0.686–0.930)	0.821 (0.631–0.939)	ADC histogram analysis improves the prediction of tumour histological grade

Abbreviations: ADC, apparent diffusion coefficient; AUC, area under the curve; MRI, magnetic resonance imaging; Sens, sensitivity; SNSCC, sinonasal squamous cell carcinoma; Spec, specificity.

### 3.6 Performance of Signatures

Model performance, estimated using the receiver operating characteristic curve and summarised as the AUC, ranged between studies. Median AUC, sensitivity and specificity across included studies were 0.947 (0.63–0.973), 0.86 and 0.923, respectively, in the training set and 0.914 (0.702–0.838), 0.833 and 0.878, respectively, in the validation set. Table 3 illustrates the performance of each model in predicting their oncological outcome. Signatures developed to predict early relapse of SNSCC had good performance, with AUCs ranging from 0.597 to 0.98. Regarding distinguishing low‐grade and high‐grade SNSCC, model performance in one study was excellent in predicting this outcome within their training cohort (AUC, 0.947).

**FIGURE 2:**
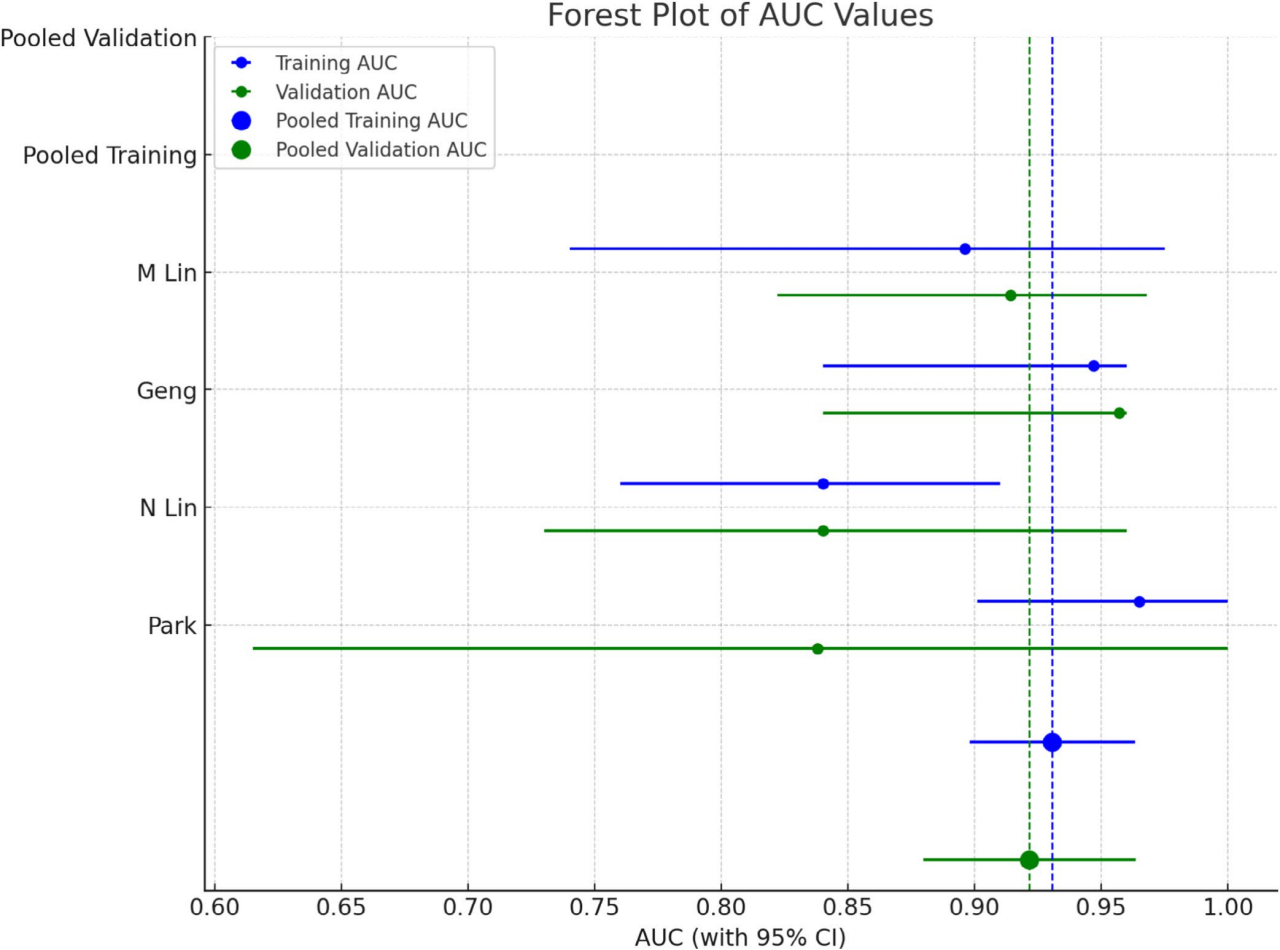
Forest plot of AUC values for training and validation sets across multiple studies.

In the pooled meta‐analysis of the AUC values, the combined estimate for the training sets across the four studies yielded a pooled AUC of 0.931 (95% CI, 0.898–0.963). Similarly, the pooled AUC for the validation sets was 0.922 (95% CI, 0.880–0.964) (Figure 2). These results indicate strong predictive performance across the studies, with relatively narrow CIs reflecting consistent findings in both the training and validation sets.

## 4 Discussion

The findings of this systematic review underscore the potential of radiomics as a predictive tool for post‐operative disease recurrence in SNSCC. Despite the relative rarity of SNSCC, the high recurrence rates post‐surgery necessitate the development of advanced prognostic tools to better stratify patients according to their risk and tailor treatment strategies accordingly [17, 27]. Radiomics, which involves the extraction and analysis of large amounts of quantitative imaging data, has emerged as a promising approach to achieve these goals [22, 23, 24, 25, 26].

The reviewed studies consistently demonstrate that radiomic signatures have satisfactory predictive performance, with median AUC values of 0.84 in both training and validation cohorts [22, 23, 24, 25, 26]. This level of accuracy is noteworthy, especially considering the inherent challenges of predicting outcomes in rare malignancies like SNSCC, suggesting that combined models may be more effective in identifying high‐risk patients who may benefit from more aggressive post‐operative treatment or closer surveillance [24, 25, 26].

However, the studies included in this review also highlight several limitations that must be addressed before radiomics can be fully integrated into clinical practice. First, the retrospective nature and single‐centre design of most studies limit the generalisability of the findings [22, 23, 24, 25, 26]. The variability in radiomic feature extraction methods and the lack of standardised imaging protocols further complicate the reproducibility and comparability of results across different studies. For instance, while some studies reported excellent performance with AUCs close to 1.0, others showed much lower predictive accuracy, indicating potential inconsistencies in the methodologies used [22, 23, 24, 25, 26].

The observation that all studies included in this systematic review were conducted in Asia is particularly intriguing, especially considering the prevalence of sinonasal cancer in this region. Sinonasal cancer, though rare globally, shows a higher incidence in certain parts of Asia, likely due to regional environmental factors, occupational exposures and genetic predispositions. The concentration of radiomics research in Asian populations may reflect an increased clinical focus on this malignancy in regions where it poses a more significant public health challenge.

Additionally, while the QUADAS‐2 and RQS tools indicated that the methodological quality of the included studies was generally satisfactory, the RQS scores above 30% suggest room for improvement [19, 20]. Higher RQS scores would indicate more robust radiomic analysis and a greater likelihood of the findings being applicable in a clinical setting [20]. Moreover, the moderate positive correlation between training and validation AUCs, although not statistically significant, suggests that while the models are relatively stable, there may be some overfitting to the training data, which could affect their performance in broader clinical applications.

The promising results of this review indicate that radiomics could significantly enhance personalised medicine in sinonasal cancer by enabling more precise risk stratification and informing treatment decisions. However, the transition from promising research findings to clinical application requires addressing the current limitations through more rigorous, multicentre prospective studies. Such studies should aim to standardise radiomic feature extraction and imaging protocols, ensuring that predictive models are both reproducible and generalisable across different patient populations. Future research should also explore the integration of radiomics with other emerging biomarkers, such as genetic and molecular data, to develop comprehensive prognostic models that could offer even greater predictive power [6, 28]. In summary, while radiomics holds substantial promise in predicting disease recurrence in SNSCC, further validation and methodological refinement are essential to unlock its full potential in clinical practice.

## 5 Conclusion

This systematic review highlights the promising role of radiomics in predicting post‐operative disease recurrence in sinonasal cancer, demonstrating its potential to enhance personalised treatment strategies. The studies reviewed consistently showed that radiomic‐based models, particularly when integrated with clinical data, offer substantial predictive accuracy, with median AUC values reaching 0.84 in both training and validation cohorts. These findings suggest that radiomics could be a valuable tool in identifying high‐risk patients and tailoring therapeutic approaches accordingly.

However, the current evidence is primarily based on retrospective, single‐centre studies, which may limit the generalisability of the findings. The variability in radiomic feature extraction methods and the absence of standardised imaging protocols further highlight the need for more rigorous, multicentre prospective studies. Future research should focus on validating these preliminary findings in larger, diverse cohorts, refining radiomic methodologies and integrating them with other emerging biomarkers to build robust, clinically applicable predictive models.

## Author Contributions

Conceptualisation and methodology: C.W., H.C.T., N.J.O. Formal analysis, investigation, data curation and writing – original draft preparation: C.W., T.H., H.C.T., N.J.O. Writing – review and editing: H.J., T.S. Supervision: T.S. All authors have read and agreed to the published version of the manuscript.

## Conflicts of Interest

The authors declare no conflicts of interest.

## Supporting information


**Data S1:** Supporting Information.

## Data Availability

The data that support the findings of this study are openly available in PubMed at https://pubmed.ncbi.nlm.nih.gov.
